# HIV and Human Coronavirus Coinfections: A Historical Perspective

**DOI:** 10.3390/v12090937

**Published:** 2020-08-26

**Authors:** Palesa Makoti, Burtram C. Fielding

**Affiliations:** Molecular Biology and Virology Research Laboratory, Department of Medical Biosciences, University of the Western Cape, Cape Town 7535, South Africa; 3062559@myuwc.ac.za

**Keywords:** coronaviruses, HIV, COVID-19, SARS-CoV-2, MERS-CoV, immunosuppression, immune response, coinfection

## Abstract

Seven human coronaviruses (hCoVs) are known to infect humans. The most recent one, SARS-CoV-2, was isolated and identified in January 2020 from a patient presenting with severe respiratory illness in Wuhan, China. Even though viral coinfections have the potential to influence the resultant disease pattern in the host, very few studies have looked at the disease outcomes in patients infected with both HIV and hCoVs. Groups are now reporting that even though HIV-positive patients can be infected with hCoVs, the likelihood of developing severe CoV-related diseases in these patients is often similar to what is seen in the general population. This review aimed to summarize the current knowledge of coinfections reported for HIV and hCoVs. Moreover, based on the available data, this review aimed to theorize why HIV-positive patients do not frequently develop severe CoV-related diseases.

## 1. Introduction

With the rapid advancement of molecular diagnostic tools, our understanding of the etiological agents of respiratory tract infections (RTIs) has advanced rapidly [1]. Researchers now know that lower respiratory tract infections (LRTIs)—caused by viruses—are not restricted to the usual suspects, such as respiratory syncytial virus (RSV), parainfluenza viruses (PIVs), adenovirus, human rhinoviruses (HRVs) and influenza viruses. Interestingly, the other causative viral agents typically have similar seasonal incidence and clinical presentations as the more “common” viruses [2,3].

With the third deadly human coronavirus (hCoV)—SARS-CoV-2 (Severe acute respiratory syndrome coronavirus-2 or SARS-2)—recently identified in China [4,5], the role of coronaviruses (CoV) in respiratory tract infections is once again under the spotlight. Human CoVs were first isolated and identified in the 1960s, first hCoV-OC43 (OC43) [6] and then hCoV-229E (229E) [7]. Only after the global outbreak of severe acute respiratory syndrome (SARS) was linked to the novel SARS-CoV [8], did the attention shift back to the hCoVs. Since then, researchers have identified NL63-CoV (NL63) [9], HKU1-CoV (HKU1) [10], and MERS-CoV (Middle Eastern respiratory syndrome coronavirus, or MERS) [11,12] as agents of both upper respiratory tract infections (URTIs) and LRTIs. 

In most cases, infection with the four “common” HCoVs (229E, OC43, NL63 and HKU1) causes mild cold-like symptoms, involving the upper respiratory tract. A severe LRTI can, however, develop in immunocompromised patients and children [13]. Unlike the common hCoVs, the more pathogenic SARS, MERS and SARS-2 frequently cause more severe LRTI. In some patients, the infection is accompanied by a cytokine storm, which can lead to a poor prognosis [14]. 

For a long time, researchers have looked at a clinical syndrome as the result of an infection by a single virus. Now we know that, even though viral interference (one virus competitively suppresses the replication of other coinfecting viruses) is the most common outcome of viral coinfections, these types of infection have the potential to influence the resultant disease pattern in the host [15]. 

Whereas, “coinfection” refers to the simultaneous infection of a cell or host by separate viruses [16], “superinfection” refers to a scenario where one virus infects the host some time before infection by the second virus. For both of these, the fate of the virus-infected host often depends on the balance between the host’s protective immunity and immunopathology [17,18]. In the context of human immunodeficiency virus (HIV) infections, however, superinfections refer to the phenomenon where an HIV infected person is infected with a subsequent distinct HIV viral strain [19]. Therefore, this manuscript will use the term “coinfection” when referring to the relationship between HIV and hCoVs in the infected host.

This review aimed to summarize the current knowledge of coinfections reported for hCoVs and HIV. Also, based on the data available, we aimed to theorize why HIV-positive patients do not frequently develop severe CoV-related disease.

## 2. HIV-hCoV Coinfections

Due to the lack of published academic work for HIV/hCoV coinfections, not much is known about the clinical and epidemiological outcomes in patients with HIV and hCoV coinfections. It is not clear whether this lack of published data is due to the lack of screening for the hCoVs, or whether the hCoVs are just not present frequently in HIV-positive patients. During the current coronavirus pandemic, this lack of information is a concern in countries with high HIV cases, especially in Sub-Saharan Africa—where 70% of people living with HIV are found [20].

Historically, the four common hCoVs are also known to cause gastrointestinal problems in HIV-positive individuals. Unfortunately, since this was before the advent of sophisticated diagnostic tools, electron microscopy was used to identify the presence of coronavirus-like particles (CVLPs) and little is known about the specific hCoVs present in the stool samples from these HIV-positive patients. Moreover, even though these are studies reporting on the findings from small HIV-positive cohorts, between 14–50% of participant’s stool samples were observed to contain CVLPs; for all of these, bar one patient, there was no correlation with enteric symptoms [21,22]. Therefore, from the late 1980s, human coronaviruses were speculated to be agents of opportunistic infections in immunocompromised hosts, and possible causative agents of diarrheal disease in AIDS [21]. More recently, it has been reported that these hCoVs can also cause more severe RTIs in HIV-positive individuals (Table 1); traditionally, CoVs were not even considered as a cause of RTIs in HIV-positive individuals [23]. 

One such study reports a 32-years old, HIV positive male, who tested positive for hCoV-229E and RSV. This patient was admitted with acute respiratory failure and died of multiorgan failure 14 days after admission to the intensive care unit (ICU). At the time of admission, the patient was not on any antiretroviral treatment (ART); he was only started on combination ART with a regimen of abacavir, lamivudine and efavirenz after testing positive for HIV [24]. 

Another study reports the detection of hCoV in HIV-positive children with LRTIs. Testing 517 samples, the authors reported the presence of different types of respiratory viruses in 60.9% of children. Interestingly, OC43, NL63 and HKU1 were detected at varying incidence in clinical samples, with 12.2%, 1.7% and 1.4%, respectively. Importantly, 70% of HIV-negative children with LRTIs also tested positive for respiratory viruses, including hCoVs [25], showing that this is a problem in all children. 

How do the three pathogenic hCoVs behave in HIV-positive patients (Table 1)? According to our knowledge, only one reported case study of an HIV-positive person infected with SARS-CoV is available. The 30-year-old male developed relatively mild symptoms, including chest pain and chills, fever, dry cough and general malaise, but recovered fully from SARS [26]. During the same period, another study reported the connection between HIV and SARS-CoV. Interestingly, despite contact between 95 SARS-confirmed patients and 19 HIV-positive individuals in a hospital ward, none of the HIV-positive patients became infected with the SARS-CoV. However, six of 28 medical personnel who worked in the same ward were infected with the SARS-CoV [27].

Researchers are now also reporting cases of HIV/SARS-CoV-2 coinfection. A study of a 61-year-old male from Wuhan, China is one of the first cases reported; this patient had type II diabetes and mild lymphopenia, and is a heavy smoker. The patient was placed on oral therapy with the anti-HIV drug, lopinavir/ritonavir on admission. He recovered fully and was discharged 20 days after first reporting to the local fever clinic [28].

A group from Spain, reports HIV/SARS-CoV-2 coinfections in five individuals—three male and two transgender patients. When the patients presented to the hospital, two had URTIs, and three viral pneumonia. Furthermore, one patient had hypothyroidism, and another asthma as co-morbidities. On admission to hospital, four of the patients were on HIV ARTs; two were using a protease-inhibitor (darunavir-boosted cobicistat), and two were using an integrase-inhibitor (dolutegravir). Additionally, all five patients were started on SARS-CoV-2 boosted-protease inhibitor ART on the day of coronavirus disease-19 (COVID-19) diagnosis. Initially, two patients were admitted to ICU, but one recovered and was released from ICU; the remaining patient remains in ICU. So, at the time of reporting, four of the patients recovered and were discharged [29]. 

An observational prospective study from Spain (Madrid) reports 51 (eight women, 43 men) SARS-CoV-2-positive cases (35 laboratory-confirmed; 16 suspected cases) among a cohort of 2873 HIV-positive individuals (an incidence 1.8%). Interestingly, these HIV/SARS-CoV-2 patients presented similar clinical, laboratory and radiographical features reported for HIV-negative patients infected with SARS-CoV-2. The authors reported that among HIV-positive individuals, those with COVID-19 has a significantly higher prevalence of co-morbidities (32 or 63%, mostly hypertension and diabetes) compared with those without COVID-19 (495 or 38%). For this cohort, 37 (73%) patients were receiving nucleoside reverse transcriptase inhibitors (NRTIs), and 11 (22%) had previous protease inhibitor treatment before COVID-19 diagnosis. The SARS-CoV-2-positive cohort included 12 mild, 38 mild-to-moderate and 13 severe COVID-19 cases. At the time of reporting, 44 COVID-19 patients recovered, six patients were still critically ill, and two patients died [30]. 

Another study from Wuhan, China looked at 1174 HIV-positive people. This group reports eight (seven males; one female) COVID-19 symptomatic patients, of which six were laboratory- and two clinically-confirmed. On admission, all eight HIV/SARS-2 patients were on ARTs (NRTIs and Non-Nucleoside Reverse Transcriptase Inhibitors). Of the eight patients, six had mild symptoms; two had severe symptoms, resulting in one fatality. Unfortunately, this group does not report any co-morbidities for the eight patients. Interestingly, another nine HIV/AIDS patients had close contact with laboratory-confirmed COVID-19 patients, but only one of them tested positive for COVID-19; this person was asymptomatic for COVID-19. At the time of reporting, this patient had Kaposi’s sarcoma and was receiving chemotherapy [31]. 

A group from Turkey studied 1224 HIV-positive individuals. They report only four HIV/SARS-CoV-2 coinfected male patients, of which three had no co-morbidities and made a full recovery. All three patients were on HIV ARTs and developed mild COVID-19 symptoms. The fourth patient, a 44-year-old with co-morbidities, including hypertension, diabetes, chronic obstructive pulmonary disease (COPD) and obesity, died. This patient was on ARTs, his HIV viral load was low, and his CD4^+^ cell count was high before he contracted COVID-19 [32]. 

Zhao et al., reports the case of a 38-year-old man with fever, accompanied by muscle aches, who was admitted to hospital in Shenzhen, China. The patient had a travel history to Wuhan during the SARS-CoV-2 outbreak, but no other COVID-19 symptoms were observed on physical examination. Computerized tomography (CT) scans showed right lower pneumonia, for which oseltamivir and interferon (IFN)-alpha inhalation were administered. He had a normal white blood cell, lymphocyte and platelet count. Even though the patient tested negative for SARS-CoV-2 by reverse transcription-polymerase chain reaction (RT-PCR) at different time points, he tested positive for the presence of SARS-CoV-2-specific antibodies 40-days later. On admission to hospital, the patient was using antiretrovirals (lamivudine, tenofovir, and efavirenz) for an HIV/Hepatitis C (HCV) coinfection. His close contact partner, also with HIV-1 infection, who was also taking anti-HIV agents, tested negative for SARS-CoV-2 RNA throughout the study [33]. 

The first reported case of HIV/SARS-CoV-2 coinfection from sub-Saharan Africa (specifically Uganda), involves a 34-year-old HIV-positive woman. On the day of admission, the patient reported a recent travel history to Italy, but was otherwise healthy and reported no COVID-19-associated symptoms. Even though the patient showed no respiratory symptoms, she developed gastrointestinal symptoms. Then, three days after admission, she developed a headache, chest pain, anorexia and muscle aches, but still no cough or shortness of breath. Prior to contracting COVID-19, she was using ARTs (tenofovir disoproxil fumarate, lamivudine and efavirenz) for five years and she had no other recorded co-morbidities. The patient made a full recovery and was discharged on day 24 [34].

In one of the largest clinical case studies describing the clinical characteristics and outcomes in HIV/SARS-CoV-2 patients—from New York City (USA)—2159 adult patients (older than 18 years old) with laboratory-confirmed COVID-19 were admitted to hospital in a one-month period. Thirty-one of these patients (or 1.4%) were HIV-1 positive. In the HIV/SARS-CoV-2 cohort, at least one co-morbidity was observed in 22 (71%) patients, with hypertension in 21 (67.7%), diabetes mellitus in 13 (41.9%) and obesity in nine (33.3%) patients. Moreover, eight (25.8%) were diagnosed with asthma or COPD. Before hospital admission, all patients were on ARTs, with an integrase inhibitor-based triple therapy used by 20 patients. Interestingly, COVID-19-associated symptoms were similar to the commonly reported ones for the HIV-negative population, with 23 patients (74.2%) presenting with fever or developing fever following admission. For this group, one patient developed mild, two moderate, 21 severe, and seven critical symptoms. At the time of reporting, eight patients had died (four had “do not resuscitate” orders), but 21 (67.7%) recovered and were discharged—13 (41.9%) home and eight (25.8%) to a care facility—and two were still hospitalized. All eight mortalities were older than 50 years old, and seven of the eight were using a tenofovir prodrug as part of their ART treatment [20]. Similarly, a large study of 5700 COVID-19-positive patients admitted to a network of New York City hospitals reports only 48 HIV-positive patients (prevalence of 0.8%). Unfortunately, not much is reported for these patients as their HIV status was merely reported as a co-morbidity [35]. 

Gervasoni et al. (2020) describe 47 HIV-positive patients with laboratory-confirmed (28, including one asymptomatic patient) or probable (19) SARS-CoV-2 infection; these were identified out of 6,000 HIV-positive patients. Almost 64% of the 47 HIV/SARS-CoV-2 patients had at least one co-morbidity (mainly dyslipidemia, arterial hypertension and Hepatitis B virus (HBV) or HCV coinfections). At the time of hospitalization, 38 of the patients were on an integrase inhibitor-based ART and 5 a protease inhibitor-based treatment; 20 were receiving a tenofovir-based regimen. The COVID-19 diagnosis of the probable cases was based on their clinical symptoms and the presence of risk factors (mainly being healthcare workers or contact with confirmed COVID-19 patients). Thirteen of the 28 laboratory-confirmed SARS-CoV-2 positive patients were hospitalized, six with severe lung disease, of which two required mechanical ventilation. At the time of reporting, 45 patients made a full recovery. Two fatalities were reported: A 47-year-old overweight man without other co-morbidities, and another patient with confirmed cardiovascular disease and a recent diagnosis of lung cancer [36]. 

Benkovic et al. (2020) report the case studies of four HIV/SARS-CoV-2 coinfected patients. All four patients had uncomplicated cases of SARS-CoV-2 infection, with symptoms commonly reported by the general population, including fatigue, loss of taste and smell, fever and cough. Co-morbidities were recorded for all four, with atrial fibrillation, hyperlipidemia, hypertension, type II diabetes mellitus and treated HCV listed. At the time of testing, all four patients were using ARTs (four were using NRTIs, three were using integrase strand transfer inhibitors, one was using a CCR5 antagonist, one was using non-nucleoside reverse transcriptases, and one was using a CYP3A inhibitor) and all had robust CD4^+^ T cell counts. Three of the patients were asked to home self-isolate, and the fourth was hospitalized; the latter was also positive for an influenza A test. At the time of reporting, all four patients recovered from COVID-19 [37].

**Table 1:** Human coronavirus infections in HIV-positive patients.

Study Cohort	hCoV (% Incidence)	Clinical Presentation	Other Types of Drugs Administered (Number Receiving the Drug)	Disease Outcome at the Time of Reporting	Co-Morbidities	Other Pathogens	Reference
Thirty-two-year old male	229E	Acute respiratory failure, respiratory distress, cough, fever, tachypnea, low CD4^+^ count, high HIV RNA count, pneumonia, acute renal failure, anemia, thrombocytopenia, elevated C-reactive Protein levels	(ART) NRTIs, antibiotics, high dose norepinephrine infusion	Fatal multi-organ failure	None reported	RSV, *E. coli*, *Proteus mirabilis*	[24]
Five hundred and seventeen samples from children	OC43 (12.2%); NL63 (1.7%); HKU1 (1.4%)	Cyanosis (11.4% vs. 8.1%), CXR-AC (Pneumonia—26.6% vs. 22.1%), C-reactive protein (CRP) levels (15 vs. 12mg/mL), fever	PCV9 (pneumococcal vaccine)	Not reported	None reported	hRV (31.7%), human bocavirus (9.5%), polyomavirus WUPyV (8.9%) Bacteria	[25]
Thirty-year old male	SARS-CoV	Low CD4^+^ count, high HIV RNA count, dry cough, fever and malaise, pneumonia, tachypnea, lymphopenia,	(HAART) NRTIs, Protease inhibitors, *Pneumocystis carinii* pneumonia prophylaxis, Ribavin + prednisolone (anti-SARS), anti-TB treatment	Full recovery	TB	HBV	[26]
Sixty-one-year old male	SARS-2	Dry cough, lymphopenia, pneumonia, dypsnea	Aloglibtin + Metformin, protease inhibitors, antibiotics, immunosuppresants	Full recovery	Type II diabetes	None reported	[28]
Three cisgender men, 2 transgender people	SARS-2	Fever, low CD4^+^ count (1/5), high HIV RNA load (1/5), elevated CRP levels (4/5), elevated ferratin levels (3/5), lymphocytopenia (2/5), thrombocytopenia, cough, LRTI (3/5), URTI (2/5), cough	ARTs, hydroxychloroquine (4), IFN beta-1b (2), antbiotic, corticosteroids (2), immunosuppresants (1)	Four recovered; 1 in ICU	Hypothyroidism (1), asthma (1)	None reported	[29]
Fifty-one COVID-positive: Eight women, 43 men (from 2873 HIV-positive patients)	SARS-2	Common symptoms: Non-productive cough, fever, dyspnea, fatigue	NRTIs (37), protease inhibitor treatment (11) hydroxychloroquine (30), azithromycin (19), ritonavir-boosted lopinavir (14)—usually used in combination, boosted darunavir (8)	Forty-four recovered; 2 fatalities	Six-three percent at least one co-morbidity (hypertension, high BMI, diabetes, chronic kidney disease, chronic liver disease)	None reported	[30]
Eight patients (from 1174 HIV-positive patients)	SARS-2	Low CD4^+^ counts (2/8—100–350 cells/mm^3^), normal CD4^+^ counts (6/8—>350 cells/mm^3^)	ARTs	One fatality	None reported	None reported	[31]
Four male patients (from 1224 HIV-positive patients)	SARS-2	High HIV RNA load (3/4), low CD4^+^ count, pneumonia, cough, lymphocytopenia, elevated ferritin levels (1/4), elevated CRP levels (2/4), diarrhoea, thrombocytopenia	ARTs (NRTIs and protease inhibitors), antibiotics	Three recovered; 1 fatality	Bipolar disorder, diabetes, COPD, hypertension, obesity	HBV	[32]
Forty-eight HIV-positive (from 5700 COVID-19 positive)	SARS-2	None reported	None reported	None reported	None reported		[35]
Thirty-eight-year-old male	SARS-2	Fever, muscle aches, fever, pneumonia, slightly elevated CRP levels, normal WBC and lymphocyte count	ARTs NRTIs, Oseltamivir, IFN-α	Full recovery	None	HCV	[33]
Thirty-four-year-old woman	SARS-2	gastrointestinal symptoms, headache, chest pain, anorexia and muscle aches	Tenofovir disoproxil fumarate, lamivudine and efavirenz	Full recovery	None	None reported	[34]
Thirty-one HIV-positive (from 2159 COVID-19 positive patients)	SARS-2	Fever viral pneumonia	All patients were on ART; integrase inhibitor-based triple therapy hydroxychloroquine (24), azithromycin (16), corticosteroids (8), IL-6R antagonist tocilizumab (2), antiviral drug—remdesivir (1), IL-6R inhibitor—sarilumab (1)	Twenty-one recovered; 8 fatalities	Seventy-one percent at least one co-morbidity (hypertension, diabetes mellitus, obesity, asthma, COPD	None Reported	[20]
Forty-seven COVID-positive (from 6,000 HIV-positive patients)	SARS-2	Fever, cough, dyspnea, diarrhea, myalgia, headache	Integrase inhibitor-based ART; protease inhibitor-based treatment; tenofovir-based regime	Forty-five recovered; 2 fatalities	Sixty-four percent at least one co-morbidity (dyslipidemia, arterial hypertension	HBV, HCV	[36]
Four male patients	SARS-2	Fatigue, loss of taste and smell, fever, cough	All patients were on ART	Full recovery	atrial fibrillation, hyperlipidemia, hypertension, type II diabetes mellitus	HCV (1) influenza A (1)	[37]

To date, studies are reporting a COVID-19 prevalence of between 0.68% [31] and 1.8% [30] in HIV-positive patients. Similar to data for HIV-negative populations, old age and co-morbidities appear to be important in the development of severe COVID-19 and mortality in HIV-positive patients [29,30,31]. Interestingly, groups are now reporting that in HIV-positive patients, the gender, the CD4^+^ counts, the HIV viral load, or the ART regimen do not show a clear association with COVID-19 infection or severity of the disease [20,29,30,31]. It appears as though, as reported for immunocompetent individuals, that co-morbidities are an important factor in mortality rates in HIV/SARS-CoV-2 coinfections. Interestingly, others report, that COVID-19 disease prognosis is improved in HIV-positive patients that are on a regular ART regimen, and where HIV viral load is suppressed [32]. 

## 3. CoV and HIV Induced Cytokine Storms

Many viral infections trigger a hyperinflammatory syndrome, resulting in cytopenias, unremitting fever, and pulmonary involvement (including acute respiratory distress syndrome (ARDS)) in about half of patients [38]. A cytokine storm is typically characterized by a decrease in inhibitory cytokines (e.g., interleukin (IL)-10, transforming growth factor (TGF)-β), an increase in activation cytokines (e.g., IL-12, interferon (IFN)-γ, TNF-α), and an increase in the infiltration of leukocytes into inflammatory sites [39]. 

In the majority of cases, HIV infection triggers an inflammatory response that presents as an acute retroviral syndrome. While this syndrome is normally self-limiting, primary HIV infection can, at times lead to a sudden and severe inflammatory process similar to cytokine storm syndrome [40,41]. Whereas, this increase in certain plasma cytokines and chemokines occurs very early after infection, cytokine storm is associated with acute HIV infection in the period leading up to peak viremia [41,42,43]. Importantly, the subsidence of the resultant cytokine storm is not complete, and if left untreated, some cytokines remain at higher than normal physiologic levels, which then persist into the chronic phase [43].

In severe lung infections, the cytokine storm-linked inflammatory response can move into the systemic circulation, producing systemic sepsis, which could result in damage of other organs [39,44]. Similarly, the three pathogenic hCoVs can also cause severe pneumonia, which is often associated with rapid virus replication, massive inflammatory cell infiltration and elevated pro-inflammatory cytokine/chemokine responses. This can then lead to acute lung injury (ALI), ARDS, and sometimes, death [14]. 

Both SARS-CoV- and MERS-CoV-linked severe disease is linked to an excessive immune response in the host, and cytokine dysregulation may account, at least partly, for the development of the severe clinical disease, ALI and ARDS [3]. SARS-CoV and MERS-CoV do not significantly stimulate the expression of certain antiviral cytokines (IFN-α and IFN-β), but do stimulate comparable levels of TNF-α and IL-6 [45]. In an in vitro Calu-3 cell system, the levels of expression of IL-1β, IL-6 and IL-8 were significantly higher in MERS-CoV infected cells than in SARS-CoV infected cells at 30 h post-infection. On the other hand, SARS-CoV induces markedly higher expression levels of TNF-α, IFN-β and IP (interferon gamma-induced protein)-10 in cells than MERS-CoV at 24 and 30 h [46]. This difference could possibly explain the difference in mortality rates reported for MERS and SARS. What could this look like?

Not only is an IFN-γ-related cytokine storm induced post-SARS-CoV infection [47,48,49], IP-10 and IL-2 are induced early in the infection. A subsequent over-production of IL-6, with a decreased production of IL-10, then likely leads to the main immunopathological damage involved in lung injury [50]. Severe pneumonia caused by MERS-CoV is also often associated with massive inflammatory cell infiltration and elevated pro-inflammatory cytokine/chemokine responses [14,45,46]. MERS-CoV induces the expression of high levels of IL-12, IFN-γ, and chemokines in the infected host [45]. 

Interestingly, a hyper-immune response is now also associated with severe COVID-19 [51]. Studies suggest that COVID-19-related mortality could also be linked to this virus-induced hyperinflammation [52], which is likely caused by a cytokine storm [53]. For severe COVID-19, the overproduction of early response proinflammatory cytokines (TNF, IL-6, and IL-1β) results in this cytokine storm. This potentially leads to an increased risk of vascular hyperpermeability, multiorgan failure, and eventually, death when the high cytokine levels are not controlled [54,55]. Why are we not seeing more frequent severe CoV-related disease, including cytokine storms, in HIV-positive individuals?

## 4. HIV-CoV Coinfections and Disease Outcome

Van der Hoek et al. noted that in respiratory coinfections, the hCoV viral load is often much lower than for single infections [56]. Even though HIV is not a respiratory virus, this could explain why the hCoV viral load in HIV-positive patients seldomly researches the levels required for the severe coronavirus-related disease to develop.

Others are now hypothesizing that the disease outcomes seen in HIV-hCoV coinfection could be due to:

- The existing infection with HIV-1 interfering with the replication of the CoV in the same host. This could result in the patient not being coinfected with the hCoV, or due to “viral interference” [15], the viral load of the hCoV remains low and severe coronavirus disease does not develop (see comments from [56] above); or
- HAART, used in the treatment of patients with HIV-1/AIDS, interferes with CoV replication, thereby preventing the development of the severe coronavirus disease [27,57]; or
- HIV induced lymphopenia could protect HIV-positive patients from severe CoV-disease clinical manifestations [58,59].

Data from human and animal studies suggest that, after infection by unrelated pathogens, the host’s immune response to subsequent infections can be altered. One virus competitively suppressing the replication of another coinfecting virus is one of the most common outcomes of coinfections [15]. Infections by certain heterologous viruses have been reported to result in protective cross-immunity by employing different processes, including innate immune activation [60], bystander protection by activated CD4^+^ or CD8^+^ T cells, and/or cross-reactive CD8^+^ T cells [15,61].

IFN-mediated innate viral interference is the most common form of one virus suppressing the replication of another, heterologous virus [62]. When IFN binds to its cognate receptor, it results in the expression of multiple interferon-stimulated genes (ISGs), which in turn control and activates various cell signaling pathways [63,64,65]. The ISGs also control the actions of numerous innate immune mediators that are able to nonspecifically block virus replication [15]. This interference can occur at various steps of virus replication, including attachment [66], entry [67], genome replication [68,69], and budding [15,70]. 

Immunity to previously encountered viruses that alters responses to unrelated pathogens is another common phenomenon amongst viruses and other microorganisms. One such example is heterologous immunity, where “exposure to one pathogen will generate an immune response against numerous antigenic epitopes derived from that pathogen, some of which might cross-react with epitopes derived from other pathogens.” When a second, unrelated pathogen infects the host, the “cross-reactive memory cells expand more rapidly and may dominate the overall response” [71]. Another case where heterologous immunity is commonly observed is in persistently infected individuals who experience constant, low-level antigenic stimulation that alters their immunity to other pathogens [60]. In one study, mice are given a modified heat-labile bacterial toxin that altered the microenvironment, such that it improved the immune response to subsequent infection with RSV, influenza virus, or the fungus *Cryptococcus neoformans*. Interestingly, this type of protective immunity is partially T cell- and B cell-independent. Cytokines produced by activated antigen-presenting cells (APCs) stimulate T cells to differentiate into polarized subsets and influence the type of immune response that is generated to a second unrelated pathogen [72]. 

Interestingly, heterologous immunity can also lead to immunopathology, instead of protective immunity. One example of heterologous immunity, resulting in immunopathology, is observed in the immunopathologic features that become exaggerated and pronounced in young adults or the elderly (also observed in COVID-19), suggesting that prior infections in young adults—that have not yet occurred in most young children—may alter the immune environment to subsequent infections in the older patients [17,73,74]. The cytokine *milieu* elicited by the active infection seems to be what drives heterologous infections, and may modulate subsequent cellular responses [17,75]. Could this explain why we are seeing a more severe pathogenic hCoV infection is some older patients? 

The reasons or mechanisms underlying the unexpected disease outcomes in HIV/hCoV coinfections are unclear. Researchers are speculating that this could be explained by the anti-HIV agents having anti-CoV properties [5], or the initial HIV infection causing an alteration of host cells so that they no longer offer a favorable environment for virus replication and/or multiplication [33], a phenomenon reported previously by Beale [76]. Moreover, researchers have speculated that for SARS-CoV—but this could probably be for SARS-CoV-2 and MERS-CoV too—the defective cellular immunity in HIV-positive patients “could paradoxically be a protective factor in some patients” [26]; another group also hypothesize that for SARS-CoV-2 “the compromised immunity might be the reason that HIV/AIDS patients did not occur inflammatory changes and clinical symptoms” [31]. Mascolo et al. hypothesize that the absence of T-cell activation in the immunocompromised patient mitigates the severe immunopathological phenomena associated with COVID-19 [77]. Based on their own data, and data from others, Shalev et al. now speculate that even “uncontrolled HIV infection and poor CD4^+^ T-cell function may limit SARS-CoV-2–related immune dysregulation and cytokine release” [20], offering some protection against the development of severe COVID-19.

## 5. Conclusions

In general, the risk associated with coinfections is still a matter of debate [78]. Therefore, with the lack of conclusive studies on HIV-CoV coinfections, researchers are concerned that the immune status that makes HIV-positive people vulnerable to other infections, could also predispose them to develop more severe CoV infections [58]. Furthermore, if we have evidence that HIV-positive people are more susceptible to develop more severe hCoV-related diseases, it could lead to the early adoption of specific therapeutic strategies [24]. 

However, the estimated COVID-19 prevalence reported in various studies does not suggest increased rates of hospitalization or mortality in HIV-positive patient populations. Clinical characteristics and disease outcomes were comparable to those described for the general population with COVID-19 [20,30,35,36]. In conclusion, recent evidence points to the fact that, even in the HIV-positive community, pre-existing co-morbidities and old age place the individual at higher risk of developing a severe disease [28,29,31]. Interestingly, the type of HIV anti-viral drug used, or the HIV viral load does not appear to be determining factors [31,58]. The veracity of all of these claims needs to be confirmed, however. 
